# The Spiritual Wellness of an Intellectual, Novelist, Journalist and Politician: The Meaningful Life of Sol Plaatje

**DOI:** 10.5964/ejop.5417

**Published:** 2021-08-31

**Authors:** Crystal Welman, Paul J. P. Fouché, Pravani Naidoo, Roelf van Niekerk

**Affiliations:** 1 Wellness Centre, Central University of Technology, Bloemfontein, South Africa; 2 Department of Psychology, University of the Free State, Bloemfontein, South Africa; 3 Department of Industrial and Organisational Psychology, Nelson Mandela University, Port Elizabeth, South Africa; University of Johannesburg, Johannesburg, South Africa

**Keywords:** Sol Plaatje, psychobiography, spirituality as life task, Wheel of Wellness model

## Abstract

The study investigates Sol Plaatje’s (1876–1932) spiritual wellness across his lifespan. He was purposively sampled due to his impact upon South African society. As an intellectual, novelist, journalist, and politician, Plaatje was also a founder member of the South African Native National Congress, which later became the African National Congress. His life history reflected a significant degree of spiritual wellness, which was uncovered through the systematic analysis of publicly available life-history materials, including primary and secondary sources. The Wheel of Wellness (WoW) model by Sweeney and Witmer was applied to interpret the biographical evidence of spirituality and meaning in his life. Spirituality, as the central life task of the WoW, and regarded as the most influential domain of a healthy individual, incorporates religious beliefs and other individualised aspects of meaning-making. Findings indicate that spirituality characterised Plaatje’s childhood years and continued to play a role throughout his adult years. His sense of meaning and purpose was personified in the promotion and preservation of human rights and dignity, which embraced inter-racial respect, compassion, and service to others.

Solomon (Sol) Tshekisho Plaatje was born on 9 October 1876 in the Free State province of South Africa (Molema, 2012; Willan, 2018). He was the seventh of nine children to two devout Lutheran Christians, Johannes and Martha Plaatje (Midgeley, 1997), who belonged to the royal Barolong tribe^1^1*Barolong* is a *tribe* of Setswana-speaking people from Botswana and South Africa (Ramoroka, 2009). They treasured their African identity and culture (South African History Online [SAHO], 2019). Plaatje’s mother named him after Solomon, the wise Biblical king because she believed that her son too, would one day become a wise man (Molema, 2012).

Shortly after Plaatje’s birth, his parents moved to the multi-cultural Pniel mission station, where they worked for German missionaries in charge of the Berlin Missionary Society (BMS^2^2The BMS was a German Protestant (Old Lutheran) Christian missionary society that supported work in South Africa, China and East Africa. They established the Pniel mission station on the Vaal River between modern Barkly-West and Kimberley, South Africa, in 1845. The BMS emphasised spiritual inwardness and puritanical values such as morality, hard work and self-discipline (Schoeman, 1985).; Schoeman, 1991). The mission school offered Plaatje an elementary education up to Grade 5 (Midgeley, 1997). At the age of 14, he worked as a student-teacher while receiving additional private tuition from a German reverend and his wife (Willan, 2018). They introduced him to Shakespeare and cultivated his natural gift for classical music and languages (Leflaive, 2014), while his mother, grandmother, and aunts steeped him in Setswana culture and oral tradition (Rule, 2016). He matured into a polyglot, proficient in English, Dutch, German and African languages, including Sesotho and Zulu (Lunderstedt, 2014). Plaatje’s fascination with African history, folklore, and proverbs was fittingly captured years later in his novel, *Mhudi*—the first of its kind published in English by a Black South African (Lunderstedt, 2014).

When he worked as a court interpreter in Mafeking^3^3The capital city of the North West Province of South Africa (Mbenga & Manson, 2012). from 1898 until 1910, he acted as a mediator between the English and Dutch magistrates, prosecutors and plaintiffs, and vice versa (Willan, 2018). This period involved a tumultuous political change in South Africa, including the Anglo-Boer war^4^4A war between the British and two Boer republics in South Africa (Willan, 2018). and the creation of the white-dominated Union of South Africa^5^5Britain granted dominion to the White minority over all Africans and other mixed races in the country (Ramoroka, 2009). (Odendaal, 2012). Plaatje also became a pioneer of African independent journalism. His extensive newspaper articles attacked unjust laws and racial discrimination in South Africa, which gave him a national voice and profile (Lunderstedt, 2014). In 1912, he co-founded the South African Native National Congress (SANNC) in protest of the Natives’ Land Act, which deprived Africans of the right to own land (Rule, 2016). The SANNC later changed its name to the African National Congress (ANC), the present-day ruling party in South Africa. Plaatje also travelled abroad extensively as part of the SANNC delegations to present the “native case” and published a book, *Native Life in South Africa,* in 1916 to expose the ruinous effects of the 1913 Natives’ Land Act (Willan, 2018). These acts laid roots for the later anti-apartheid movement as Plaatje embraced a vision of a joint South Africa (Rule, 2016). He advocated for a united, inclusive nation based on justice and equality, sadly at great personal cost to himself and his family (Willan, 2018).

This study aimed to demonstrate the value that spirituality, as a life task of holistic wellness within the Wheel of Wellness (WoW) model, held for Plaatje over his lifespan. Plaatje’s spiritual wellness, despite adversities, is of scientific interest in terms of how it relates to resilient living and wellbeing. Thus, this contributes to the study of meaning-creation in psychobiographical research. Plaatje remains “one of the great South Africans of the 20th century” (Lunderstedt, 2014, p. 78).

## The Life Tasks of the WoW Model: The Role of Spirituality in Personal Meaning-Making

Firmly positioned within the Positive Psychology movement, the WoW model provides a holistic, integrative approach to wellness (Myers, Sweeney, & Witmer, 2000; Sweeney & Witmer, 1991; Witmer & Sweeney, 1992). It emphasises Adler (1927, 1992) concepts related to adaptive and optimal human functioning, strengths, and coping (Myers & Sweeney, 2004, 2008). The WoW model’s multi-dimensional nature explores the expression of personal wellness as a function of five interrelated life tasks as they interact with external life forces, which are, in turn, influenced by the broader context of global events (Myers et al., 2000; Roscoe, 2009). Spirituality is conceived as the central life task of the WoW model, further integrating the life tasks of self-direction (previously called self-regulation), work and leisure, friendship, and love (Myers & Sweeney, 2004; Shannonhouse, Myers, & Sweeney, 2016). The life forces include family, community, religion, education, government, media and business or industry, while global events include natural or human-made disasters, such as war, pollution, and poverty (Myers et al., 2000; Witmer & Sweeney, 1992).

The multi-dimensional concept of spirituality is considered a creative energy source for purposiveness in life (Sweeney & Witmer, 1991), which is distinguishable from the institutionalised concept of religiosity (Mayer, Van Niekerk, & Fouché, 2020). Spirituality, as described by Myers et al. (2000, p. 9) is “an awareness of a being or force that transcends the material aspects of life and gives a deep sense of wholeness or connectedness to the universe,” with a focus on “life-enhancing beliefs about human dignity, human rights and reverence for life” (Witmer & Sweeney, 1992, p. 141). The seven “subtasks” of the life task of spirituality include: 1) belief in God as a higher power; 2) religious choice and practices; 3) transcendence; 4) meaning and purpose in life; 5) hope and optimism; 6) values; and 7) love, compassion and service towards others (Myers et al., 2000; Witmer & Sweeney, 1992). Within the WoW model, optimism refers to “… an expression of hope that, with a certain degree of confidence, one can either expect the best possible outcome or dwell on the most hopeful aspects of the situation” (Witmer & Sweeney, 1992, p. 141). Transcendence is an individual’s need to attain inner peace and experience a sense of oneness with nature and the universe or the infinite (Myers et al., 2000), which is another core component in the life task of spirituality (Witmer & Sweeney, 1992). Transcendence supposedly increases life satisfaction, which, in turn, promotes psychological wellness (Toner, Haslam, Robinson, & Williams, 2012). Furthermore, in conjunction with optimism and inner harmony, moral and ethical values may also result in guiding an individual’s behaviour toward the promotion of the common good, love, compassion, and service to others (Sweeney & Witmer, 1991). Studies have indicated that individuals with a sense of purpose and those that demonstrate altruistic behaviour tend to experience a higher sense of meaning and wellbeing in their lives (Aknin, Dunn, & Norton, 2012; Mosak & Maniacci, 2008), which also cultivates future resilience to adversity (Diener & Seligman, 2004; Steger, 2012).

Ultimately, spirituality is the core dimension or life task to be developed for it to positively influence the development of the rest of the life tasks in the WoW model (Myers & Sweeney, 2008; Sweeney, 2009). Personal meaning greatly relates to spirituality (Fouché, Burnell, & Van Niekerk, 2015), and also endorses a sense of oneness, inner life, purposiveness, optimism and values.

## Research Aim

The purpose of this study is to contribute to psychobiographical research on meaning-creation. More specifically, the study aims to explore spirituality as the central life task of Plaatje within significant historical periods over his lifespan and the impact it had on his ability to consistently create meaning through the lens of the WoW model as postulated by Myers et al. (2000).

## Method

### Design and Subject

Psychobiography aims to explore and describe the development, growth, creativity, and productivity of distinguished, extraordinary and even controversial individuals over their entire lifespan, within their socio-historical contexts and from a psychological frame of reference (Fouché & Van Niekerk, 2010; Ponterotto, 2015; Ponterotto & Reynolds, 2017). Therefore, this qualitative, longitudinal study, with its morphogenic perspective, utilised a psychobiographical single case study research design and methodology (Flick, 2006; Köváry, 2018; Ponterotto, 2014; Roberts, 2002). The psychobiographical subject, South African born anti-apartheid activist, journalist, and novelist, Sol Plaatje (1876–1932), was selected through non-probability purposive sampling. A leading figure in South Africa’s liberation history, Plaatje is best remembered as one of the founding members of the 1912 SANNC, the forerunner of the present-day ANC. Plaatje lived through times of tumultuous political change, yet he consistently demonstrated tenacity and resilience, often under very trying conditions (Willan, 2018).

Plaatje’s personal commitment and contribution to the establishment of democracy in South Africa might be described as acts of “lifelong heroism,” which, according to Franco, Blau, and Zimbardo (2011) may be seen as the pinnacle of an exemplary life. The researchers contend that a renewed focus on anti-apartheid activists might contribute to a deeper understanding of South Africa’s troubled past by exploring these individuals’ roles in the journey towards democracy. This, combined with a personal interest in his unique and controversial personality, served as the rationale for selecting Plaatje as psychobiographical subject. Furthermore, despite the wealth of information available on Plaatje, a documented in-depth psychological analysis of his life did not exist at the time of undertaking this study.

Posthumously, Plaatje was awarded an array of accolades, including an honourary doctorate degree from the University of the North West in 1998 (Willan, 2018), for his humanitarian conduct and contribution to the anti-apartheid struggle. His 1916 classic, *Native Life in South Africa*, was republished in 2007 and continues to inform debates about the struggles of the African people who were relegated to landless and dispossessed squatters by the Natives’ Land Act of 1913 (Jaffer & Tshabalala, 2014). Other tributes include the following (Jaffer & Tshabalala, 2014; Willan, 2018):

1. Plaatje's home in Kimberley was declared a national monument in 1992 and is currently operating as the Sol Plaatje Library and Museum.
2. His grave in Kimberley’s West End Cemetery was declared a provincial heritage site in 1999.
3. The Kimberley Municipality was renamed Sol Plaatje Local Municipality in 1995.
4. In 2000, the ANC initiated the Sol Plaatje Award, which annually recognises the best performing ANC branch.
5. The Sol Plaatje Secondary School in Mafikeng opened its doors in 2002.
6. The Sol Plaatje University in Kimberley officially opened its doors in 2014.
7. On 8 January 2020, South African President Cyril Ramaphosa honoured Plaatje’s memory at his gravesite in the West End Cemetery during an official visit as part of the 108th anniversary celebrations of the ANC.

### Data Collection, Extraction, and Analysis

The authors retrieved numerous published documents. Primary sources included a wide range of newspaper articles, books, public speeches, and extracts from letters written by Plaatje. His politically-based book, *Native life in South Africa*, his romance novel, *Mhudi,* and *Anglo-Boer War diary*, provided valuable personal information about the subject. Secondary sources included written biographical accounts (e.g., Midgeley, 1997; Mokae, 2012; Molema, 2012; Pampalis, 1992; Rall, 2003; Willan, 1984, 2018), as well as audio-visual documentaries (e.g., Couzens, 2001; Couzens & Willan, 1979). Additional journal articles, literary essays and critiques on Plaatje ensured data triangulation that enhanced the study’s trustworthiness and rigour (Welman, Fouché, & Van Niekerk, 2019).

Important biographical data were extracted via Alexander’s (1988, 1990) nine well-known indicators of salience. Also, the WoW model (Myers et al., 2000; Sweeney & Witmer, 1991; Witmer & Sweeney, 1992) was utilised to conceptualise and uncover Plaatje’s spiritual wellness or spirituality as central life task. The authors ensured the systematic extraction and analysis of salient biographical material collected from across five significant historical periods in his life, namely: The Formative Years: Doornfontein and Pniel (1876–1894); Plaatje at Kimberley (1894–1898); Plaatje at Mafeking (1898–1910); Tales of Travels (1910–1923); and The Autumn Years (1923–1932).

### Ethical Considerations

Permission to conduct this study was granted by the Faculty’s Institutional Review Board (IRB), the Committee for Title Registrations within the Faculty of the Humanities at the University of the Free State. Relevant written consent to undertake this research, and access to unpublished archival documents, were granted by Plaatje’s family members and the director of the Sol Plaatje Educational Trust. All information was treated with respect, and best ethical practice was informed by the decision-making guidelines as prescribed by Ponterotto (2013, 2014, 2015, 2017) and Ponterotto and Reynolds (2017, 2019). Following a contextualised approach to ethical decision-making, including personal and professional reflexive research practice (e.g., reflective journalling, regular consultations with knowledgeable colleagues) and rigorous methodological checks (Ponterotto, 2014; Willig, 2013; Yin, 2018), served to enhance the trustworthiness and rigour of the study.

## Findings and Discussion

The findings pertaining to spirituality as life task (i.e., spiritual wellness) are presented and discussed chronologically according to five significant historical periods throughout the lifespan of Plaatje.

### The Formative Years: Doornfontein and Pniel (1876–1894)

Plaatje’s family of origin and family-like community cultivated particular values in him, especially concerning spirituality and faith. As the son of devout Lutheran Christians, religious worship formed a significant part of his upbringing (Mokae, 2012; Willan, 1984, 2018). Religious practices within the Plaatje household (e.g., Bible study and prayer) attest to his early exposure to the concept of God as a “higher power.” His father and brother’s respective involvement in the church as deacon and elder also affected Plaatje’s consciousness as he matured (Willan, 2018). The Pniel residents’ work ethic, diligence, and impeccable behaviour highlighted the values of the Christian faith and philosophy and heightened Plaatje’s awareness to adhere to this “higher power” and certain moral and ethical obligations (Molema, 2012; Willan, 2018). The racially-integrated environment at Pniel and exposure to the values of the Christian faith, in combination with the beliefs and customs of his African identity, further contributed to the development of values such as respect for diversity and human dignity, which, according to Myers et al. (2000), are important aspects of spirituality. Findings further suggest that Plaatje derived meaning from spending time outdoors because, as an adult, he would often longingly reflect on this time, as well as the continuing impact of nature on his mood. This aspect of spirituality (i.e., transcendence) remained an important part of his spiritual wellness experience throughout his lifespan (Welman, 2020).

At the age of 16, spirituality developed into a more personal experience for Plaatje when he was formally christened into the Lutheran faith (Fromme, 2010; Midgeley, 1997). The singing of hymns also became more meaningful as he matured, reflected by his unyielding devotion to the translation thereof in subsequent historical periods (Willan, 1984, 2018). As a student-teacher, Plaatje’s popularity with children (Molema, 2012; Rall, 2003) allowed him to experience another dimension in the life task of spirituality, namely meaning and purpose. Furthermore, Plaatje’s well-mannered demeanour, combined with his optimistic and confident interpersonal attitude (Willan, 2018), laid the foundation for his future sense of compassion and dedication to being of service to others.

In terms of global events, findings indicate that the discovery of diamonds and gold in South Africa not only escalated levies and taxes that led to financial hardship on many Africans in the area, but it also intensified land and labour disputes between the Afrikaners^6^6A South African ethnic group (Willan, 2018). and the British (Midgeley, 1997; Willan, 2018). Another global event that impacted Plaatje’s spiritual wellness includes the domination of mission Christianity and its associated educational institutions on African political life (Nasson, 2004), which eventually influenced Plaatje’s parents’ decision to adopt various practices of European missionaries (Gerhart & Karis, 1977; Nasson, 2004; SAHO, 2019).

### Plaatje at Kimberley (1894–1898)

After his arrival in Kimberley^7^7The capital city of the Northern Cape Province of South Africa (Willan, 2018)., Plaatje formed a friendship with Isaiah Bud-M’belle, which integrated him with other mission-educated Africans who stayed at the Malay^8^8A name given to inhabitants originally from Malaysia and Indonesia (Willan, 2018). Camp, a racially mixed residential area (Leflaive, 2014). This African community shared Plaatje’s morals and commitment to the Christian faith, which possibly further influenced his spiritual wellness and sense of connectedness. Isaiah later became Plaatje’s brother-in-law when he married Isaiah’s sister, Elisabeth, in 1898 (Rall, 2003). Despite initial opposition from their families, Plaatje and Elisabeth’s inter-tribal union eventually gave a romantic expression to the spiritual dimension of love (Willan, 1984, 2018).

Throughout this period, Plaatje remained a faithful Christian and was involved in church fundraising campaigns and even became secretary of the Young Men’s Christian Association (YMCA) in 1896 (Midgeley, 1997). It is thus inferred that Plaatje derived a significant sense of meaning and purpose by serving others and by gaining exposure to belief systems different from those of his family of origin (Welman, 2020). His belief in a higher power and the practice of prayer and reflection remained an important part of Plaatje’s life (Welman, 2020).

While his faith remained unchanged, the exposure to the community at the Malay Camp and the broader town of Kimberley triggered questions regarding certain government policies of segregation and division, and Plaatje became more spiritually aware that a form of political dissent had started to develop within his work environment (Willan, 1984, 2018). Therefore, when he qualified to vote in the national elections mid-1898, he did so without hesitation (Midgeley, 1997; Willan, 1984). For Plaatje, it was his duty to vote since it directly impacted his life in terms of liberty, morality and ethics, and the focus on human dignity (Welman, 2020), which are all important aspects of spirituality within the WoW model (Myers et al., 2000; Witmer & Sweeney, 1992).

A global event, the Rinderpest epidemic (also called the *cattle plague*) that swept across the country in 1896 (SAHO, 2019), exacerbated the struggling economy and intensified land and labour disputes between the Afrikaners and the British (Willan, 2018). Although Plaatje’s father also died during this time, evidence in the literature suggests that it did not negatively impact Plaatje’s spiritual wellness. At the end of this historical period, Plaatje’s pursuit of a more meaningful job culminated in his appointment as a clerk/court interpreter at Mafeking’s magistrate’s court (Midgeley, 1997; Molema, 2012). However, his optimism of moving to Mafeking in 1898 was thwarted by an illness suspected to have been malaria—the first of many documented incidents of ill-health (Midgeley, 1997; Molema, 2012; Willan, 2018). Nevertheless, Plaatje was excited about becoming a father and enjoyed spending time at home with his pregnant wife (Rall, 2003). Plaatje’s marriage anchored him throughout his life and seemed to also consistently promote his spiritual wellness (Welman, 2020).

### Plaatje at Mafeking (1898–1910)

Plaatje’s religious milieu remained unchanged as the community where he stayed when he moved to Mafeking shared his moral and ethical values (Willan, 1984). A strong affiliation with the local Barolong also allowed him to experience companionship that likely elevated his degree of spiritual wellness (Welman, 2020). Plaatje was, however, affected by the government’s indifference to the plight of many poverty-stricken Africans who were struggling economically in the aftermath of the Rinderpest epidemic (Lunderstedt, 2014; Molema, 2012). It invoked in him strong feelings of compassion and a sense of moral obligation and service to these affected families (Willan, 1984, 2018). Despite obvious tensions and conflict at work, Plaatje maintained an optimistic attitude, which constitutes an important dimension of the WoW model’s life task of spirituality (Myers et al., 2000). His work as court interpreter could be seen as an expression of this spiritual dimension. The combination of optimism, inner harmony, and values may lead to the promotion of important dimensions of the life task of spirituality, such as social interest in the form of compassion and service to others (Myers et al., 2000; Sweeney & Witmer, 1991).

The Anglo-Boer war was a global event during this historical period that not only impacted Plaatje’s personal development, but it also fuelled his sense of purpose and inspired the writing of *The Boer War diary: An African at Mafeking—*a documentation of events during a painful and disturbing time (Jaffer & Tshabalala, 2014). Biblical and musical metaphors were two of Plaatje’s favourite ways of connecting his past and present experiences in these diary entries (Willan, 1984, 1996). They reflected his belief in God as a higher power despite the circumstances. He referred to Sundays as “the Lord’s day” (Plaatje, 1999, p. 50), indicating his continued participation in worship at church. Horse-riding offered him another spiritual experience (Plaatje, 1999). His supplementary job as liaison between the White British administration and the Black Barolong chiefs (Leflaive, 2014) was another opportunity for him to serve others with purpose and meaning (Welman, 2020).

Recurring incidents of ill-health throughout this period frustrated Plaatje because it disrupted his sense of inner harmony, peace, and purpose (Welman, 2020), especially when he was confined to bed and unable to work (Willan, 2018). Plaatje experienced significant spiritual growth in this historical period. Bearing witness to the increasing apartheid policies sensitised him to the effects of discrimination and persecution (Willan, 2018) and further fuelled his sense of purpose. The biased nature of the Afrikaner government also stimulated his interest in the ethical issues involved in local politics, and so he frequently attempted to engage with the Afrikaner community about the iniquities of their policies (Willan, 1996, 2018). After the war, Plaatje experienced a personal injustice when he was deprived recognition as the top candidate in the Cape Civil Service examinations^9^9Entry examinations required for recruitment and admission to government service (Willan, 2018). (Midgeley, 1997). This was especially difficult for him to accept because, since childhood, he had always derived much meaning and purpose from improving himself, particularly on an educational level, and linked his achievements to a sense of inner harmony, peace, and spiritual growth (Rall, 2003; Willan, 2018).

Changing to a career in journalism full-time in 1902 boosted his sense of hope and optimism for the future (Welman, 2020). As its editor, the Setswana-English newspaper’s political creed echoed Plaatje’s own long-standing belief in equality before the law and pride in his African heritage (Willan, 2018). Plaatje’s advocacy for educational advancement for Africans was often juxtaposed with public criticism when he disagreed with their use of alcohol, gender discrimination or enforcement of traditional customs (such as circumcision) incompatible with Christian practice (Leflaive, 2014; Willan, 2018). The media thus became a powerful influence on Plaatje’s wellness during this historical period, as it provided him a platform from which to shape public policies as leading spokesperson for African opinion (Welman et al., 2019).

Plaatje’s political involvement increased significantly during this historical period. His vehement opposition to the implementation of the new Union of South Africa, amongst other activities, earned him the position of assistant secretary of the SANNC (Willan, 2018). His dedication to serving his people reflects courage and a high degree of spiritual wellness in the dimension of love, compassion, and service to others (Myers et al., 2000).

On the home front, when Elisabeth and their first-born son, Sainty, finally moved to Mafeking, Plaatje was excited to express his love and care to his own family and not only to the broader community (Welman, 2020). He also fathered two daughters, Olive and Violet, and two more sons, Richard and Halley (Molema, 2012). However, his regular, long absences away from home came at a great personal cost as it created an imbalance between his personal acts of love to his family and the need to contribute to society (Welman et al., 2019). Setswana orthography, specifically the research of Setswana linguistics and the preservation of vernacular languages, also kept him busy (Lunderstedt, 2014; Midgeley, 1997) and added an extra element of spiritual wellness in terms of personal meaning and purpose (Welman, 2020). Unfortunately, his newspaper's demise in 1906 sparked a decline in his general sense of meaning and purpose, along with an already precarious financial situation that remained dire for almost 3 years (Lunderstedt, 2014).

### Tales of Travels (1910–1923)

Plaatje experienced a renewed sense of hope and optimism once he was back in Kimberley. The media became a significant life force that provided him a wider platform to distribute information about the struggles of the Black South African community (Shillington, 1985; Willan, 2018), thus, enabling him to better meet his goal of opposing the apartheid government. He wrote editorials, addressed and broadly participated in protest meetings mainly against the abolishment of the Cape’s non-racial franchise (Odendaal, 2012; Shillington, 1985), which relates to aspects of social connectedness and social interest (Myers et al., 2000) in the larger South African community. Through his literary endeavours, speaking engagements, and contact with people at grassroot level, he contributed towards the empowerment and education of his culture.

Two global events, World War I (Willan, 2018) and the Spanish influenza (Phillips, 1990), significantly impacted Plaatje’s generative concern, although he continued to enhance his overall experience of meaning and purpose by writing the book, *Native Life in South Africa*, in reaction to what he experienced as an extremely challenging life force, the 1913 Natives’ Land Act (Odendaal, 2012). He also capitalised on his position as secretary-general of the SANNC (later the ANC), as well as his ties to two other inter-racial organisations, the Independent Order of the True Templars (IOTT) and the Brotherhood Movement, to raise public awareness on issues regarding racial segregation in South Africa (Rall, 2003; Willan, 2018). He felt particularly connected to the values of spirituality, sobriety, self-improvement, and racial equality promoted by the IOTT and the Brotherhood Movement (Plaatje, 1916). Furthermore, Plaatje co-authored two books dedicated to the preservation of Setswana proverbs (Jones & Plaatje, 1916) and wrote a chapter in a book commemorating Shakespeare’s 300th anniversary, published in 1916 (Gollanz, 1916; Remmington, 2013). Sweeney and Witmer (1991) stated that the combination of optimism, inner harmony, and values may also promote the common good and social interest, which Myers et al. (2000) defined as the dimension of love, compassion, and service to others. Plaatje’s vast involvement in activities despite his declining health highlighted an expression of wellness in his spiritual dimension. His travels abroad during this period also reflected a significant experience of meaning and purpose particularly since it was linked to his sense of responsibility towards the preservation of human rights and dignity, morality, and ethics (Myers et al., 2000).

Plaatje’s spiritual wellness was negatively impacted when his youngest son, Johannes, and eldest daughter, Olive, died (Odendaal, 2012; Willan, 2018). Although he tried to appear as optimistic as ever in public, these events impacted his belief in a higher power, especially since financial constraints prevented him from being able to attend their funerals (Willan, 2018).

### The Autumn Years (1923–1932)

Plaatje’s family and friends continued to play an important role in his spiritual life. They formed an integral part of his support system during the periods when his sense of optimism was particularly challenged by the life forces of government and community and thus promoted his effective management of stress (Welman, 2020). His newspaper articles and temperance work under the auspices of the IOTT, as well as personal visits and interviews with political leaders to protest against additional segregation policies (e.g., the 1926 Colour Bar Act) (Lunderstedt, 2014; Rall, 2003; Willan, 2018), relate to spiritual wellness as well as concepts in the life task of love, compassion and service to others (Myers et al., 2000). This dogged dedication to the advancement of human rights, morality and civil liberties reflects that his ethical and moral values remained a prominent dimension of his spiritual life task (Lunderstedt, 2014).

The abolishment of the non-racial Cape franchise in 1930 exacerbated the dissent within the SANNC, and Plaatje resigned as its secretary-general (Jaffer & Tshabalala, 2014). South Africa’s growing apartheid system weighed heavily on him, but he maintained his trust in the potential of individuals to behave righteously (Molema, 2012). He continued to engage in activities that served a social purpose (e.g., sobriety campaigns, preserving Setswana linguistics) and, in doing so, continued to express his generative concern for the next generation of South Africans, despite persistent financial- and health-related difficulties (Willan, 2018). This behaviour reflected a dimension of spirituality that allowed him to translate his own ethical beliefs and moral convictions regarding equality and liberty into practice.

While Plaatje’s anti-apartheid work was being recognised by the international community (Lunderstedt, 2014), he was aware that his contributions were not always appreciated amongst people from his own community. Furthermore, the excessive drinking of his sons, Sainty and Halley, directly challenged his efforts to advocate sobriety (Willan, 2018), which may have negatively influenced the life task of spirituality within his own family. Plaatje’s customary belief in a power beyond himself was again reflected in this context when he noted: “blessed are the dead who die in the Lord, for they rest from their labours” (Willan, 2018, p. 462).

Plaatje’s involvement in the preservation of Setswana literature was an important theme throughout the last years of his life (Midgeley, 1997). His close personal relationships with family and friends, and his literary achievements (i.e., the Setswana translation of Shakespeare’s *Comedy of Errors* and his novel, *Mhudi*, in addition to the already published *The Boer War diary: An African at Mafeking* and *Native Life in South Africa*), facilitated a sense of meaning, purpose, and service (Myers et al., 2000). His spirit and passion were as undaunted as ever, despite struggling with ubiquitous health issues (Molema, 2012), which suggests that Plaatje still experienced a sense of hope and optimism as a dimension of the spirituality life task (Myers et al., 2000). Career-wise, Plaatje seemed to have maintained a relatively high degree of wellness in the life task of spirituality throughout his lifespan, especially regarding the dimensions of meaning, purpose, and a sense of worth (Welman, 2020). His resilience during challenging circumstances endured until the end when he, despite being critically ill and bed-ridden, braved the cold weather and attended to prior arranged work appointments (Rall, 2003). This tenacity suggests that work facilitated a significant sense of fulfilment and remained a major influence on his ability to lead a meaningful spiritual life.

### Conclusion and Recommendations

Within the WoW model, spirituality is seen as the core trait for the maintenance of spiritual health and wellbeing as it impacts positively on the other life tasks of wellness. Plaatje’s environment cultivated within him aspects of the life task of spirituality, namely: 1) moral and ethical values to guide daily living; 2) some form of worship, prayer in relation to the Infinite, and self-reflection; 3) hope and optimism; and 4) transcendence, which includes inner peace, harmony, and oneness with nature, the universe or the Infinite (Myers et al., 2000). Belief in God as a higher power, respect for diversity, and the preservation of human dignity and human rights, as well as love, compassion, and service to others, were equally prominent features of Plaatje’s life task of spirituality. His sense of meaning and purpose was personified, especially through the promotion and preservation of human rights and dignity, evident by his extensive involvement as a political activist and human rights campaigner.

Life forces and global events, including Plaatje’s reactions towards them, influenced his degree of spiritual wellness and ability to experience meaningfulness at any given stage. Despite specific and significant changes (e.g., the implementation of the 1913 Natives’ Land Act) and challenges (e.g., the death of his two children) that occurred at different points in his life, Plaatje’s response to these changes and challenges created an opportunity to fortify both his generative concern and sense of purpose. This relates to the contention that a source of generativity has been found to be a good predictor of meaningfulness (Schnell, 2011). Contextual and systemic life forces of government, community, family, and religion had the most identifiable influence on his life task of spirituality.

The study demonstrated how Plaatje’s spiritual wellness contributed to his ability to lead a meaningful life. The findings affirm the applicability of the WoW model for psychobiographical studies of extraordinary individuals. The authors suggest that future research projects exploring the relationship between meaning-creation and holistic wellness may uncover further insights that could possibly be developed into inspirational coping resources to be used with clients in counselling.
